# Simultaneous Determination of Six Acidic Herbicides and Metabolites in Plant Origin Matrices by QuEChERS-UPLC-MS/MS

**DOI:** 10.3390/molecules30040852

**Published:** 2025-02-12

**Authors:** Qiqi Jin, Qianwen Xu, Zhiyong Zhao, Wenshuai Si, Bing Bai, Lei Chen, Changyan Zhou

**Affiliations:** 1 The Institute of Agro-Food Standards and Testing Technology, Shanghai Academy of Agricultural Sciences, Shanghai 201403, China; jeanette_jin@163.com (Q.J.);; 2 Shanghai Co-Elite Agro-Product Testing Technology Service Co., Ltd., Shanghai 201403, China; 3 School of Chemical and Environmental Engineering, Shanghai Institute of Technology, Shanghai 201418, China

**Keywords:** QuEChERS, UPLC-MS/MS, acid herbicides, residue detection

## Abstract

This study presents a method for the simultaneous determination of six acidic herbicides and their metabolites in various matrices, including fruits, vegetables, grains, and edible oils. The method employs acidified acetonitrile extraction combined with dispersive solid-phase extraction cleanup (dSPE) using MgSO_4_, Florisil, and Graphitized carbon black (GCB). The analysis was performed by ultra-performance liquid chromatography–tandem mass spectrometry (UPLC-MS/MS) with electrospray ionization (ESI) in both positive and negative modes using multiple reaction monitoring (MRM). The mass concentrations of six herbicide pesticides and their metabolites were predominantly within the range of 0.0005~0.050 mg/L and exhibited strong linear relationships with the corresponding peak area, with the coefficient of determination (R^2^) exceeding 0.993. The limits of detection (LOD) for the method ranged from 0.0001 to 0.008 mg/kg. The recovery rates of adding recovery experiments to cabbage, chives, pear, wheat flour, and soybean oil were 69.8~120%, and the relative standard deviation (RSD) was 0.6~19.5%. The results indicate that this method is efficient and fast, and can be used for the detection of compounds in various actual matrices.

## 1. Introduction

**Figure 1:**
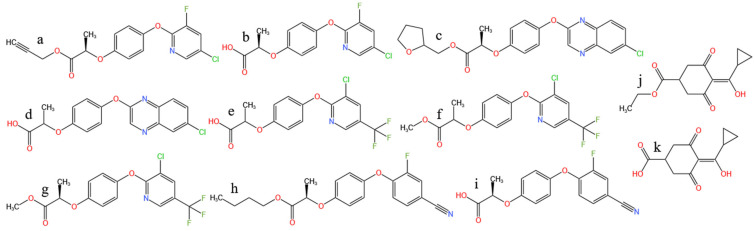
(**a**) Clodinafop-propargyl; (**b**) Clodinafop; (**c**) Quizalofop-P-tenfuryl; (**d**) Quizalpfop; (**e**) Haloxyfop; (**f**) Haloxyfop-methyl; (**g**) Haloxyfop-P-methyl; (**h**) Cyhalofop-butyl; (**i**) Cyhalofop acid; (**j**) Trinexapac-ethyl; (**k**) Trinexapac.

Clodinafop-propargyl (CP), quizalofop-P-tefuryl (QPT), haloxyfop-methyl (HM), and haloxyfop-P-methyl (HPM) are classified as phenoxypropanoic acid herbicides. The residue definition for CP encompasses both CP and its metabolite clodinafop (C). Similarly, the residue of QPT is defined as the sum of QPT and quizalofop (Q), represented as QPT. The residues of HM and HPM are defined as the sum of these compounds and their conjugates, collectively represented by haloxyfop (H). Cyhalofop-butyl (CB), an aryloxyphenoxypropionate herbicide, has a residue profile that includes CB and its metabolite, cyhalopfop acid (CA) [1]. Trinexapac-ethyl (TE), a cyclohexane carboxylic acid pesticide, was initially as a plant growth regulator [2] but also exhibits herbicidal properties [3]. Its residue is represented by its metabolite, trinexapac (T). The structural formulas of these herbicides are shown in Figure 1, all of which contain carboxyl groups and are acidic herbicides.

The registration information for six herbicides—CP, QPT, CB, TE, HM, and HPM—is increasingly available on the China Pesticide Information Network, and they have been widely used to control weeds in crops such as wheat, corn, soybeans, and potatoes [4]. However, the national standard GB 2763-2021 “National Food Safety Standard-Maximum Residue Limits (MRLs) for Pesticides in Food” [1] does not currently recommend specific detection methods for these compounds. Consequently, only temporary MRLs have been established for a limited number of agricultural products. Recent studies have investigated the residues of these herbicides in specific crops. Han Hedan et al. [5] detected the residual levels of CP in barley, while Yang Xiaolu et al. [6] analyzed the CB and CA in fruits and vegetables; there were a total of seven samples with detected target compounds, and the results were all below the EU limit. Li Yan et al. [7] determined QPT residues in potatoes. Although detection methods for these herbicides have been reported individually, no unified method has been established. Therefore, the development of a rapid and efficient detection method for these herbicides in plant origin matrices, such as vegetables, fruits, and other varieties, is essential for comprehensive reside monitoring.

In the field of herbicide residue detection, the QuEChERS procedure [8,9] is widely adopted and often coupled with gas chromatography or liquid chromatography–mass spectrometry for detection purposes [10,11,12,13]. Nevertheless, acidic herbicides are significantly influenced by environmental pH values and purification agents. Given that the pre-treatment method in the national standard GB 23200.121-2021 “National food safety standard-Determination of 331 pesticides and metabolites residues in foods of plant origin-Liquid chromatography-tandem mass spectrometry method” [14] is inappropriate for acidic pesticides, this study optimized the QuEChERS procedure. By integrating it with UPLC-MS/MS, a residual detection method for six herbicides and their metabolites in fruits, vegetables, grains, and edible oils was developed. Moreover, actual samples were analyzed using this method, Characterized by high precision and good accuracy, this method is suitable for the efficient and rapid determination of these six herbicides and their metabolites in agricultural products.

## 2. Results and Discussion

### 2.1. Optimization Results of Mass Spectrometry Parameters

**Figure 2:**
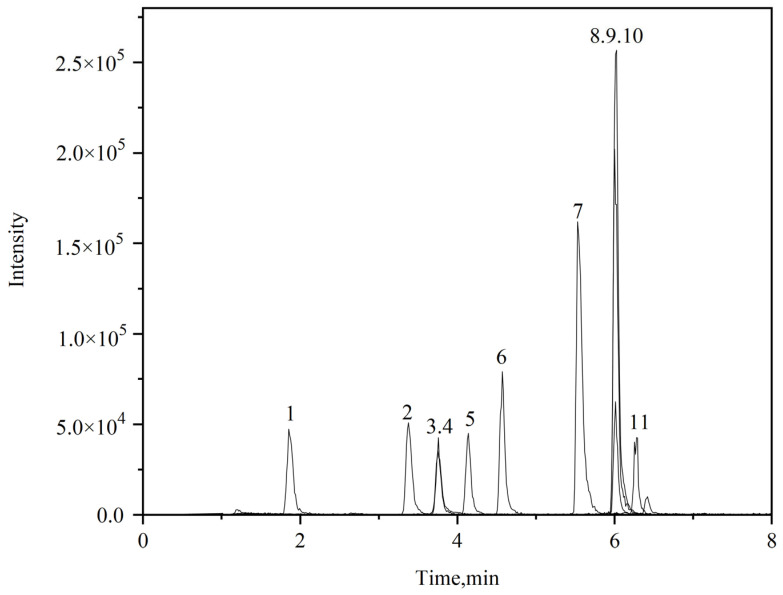
TIC of the 11 pesticides standard solution. 1, T (0.02 mg/L); 2, CA (0.02 mg/L); 3, C (0.02 mg/L); 4, TE (in) (0.05 mg/L); 5, Q (0.02 mg/L); 6, H (0.02 mg/L); 7, CP (0.02 mg/L); 8, HPM (high) (0.02 mg/L); 9, HM (mid) (0.02 mg/L); 10, QPT (low) (0.02 mg/L); 11, CB (0.1 mg/L).

**Figure 3:**
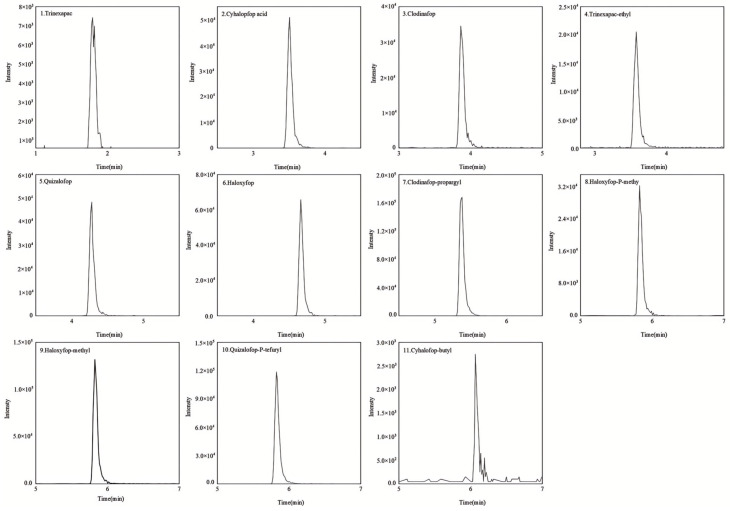
Quantitative extracted ion chromatograms of 11 pesticides standard. (The concentration of the compounds is consistent with Figure 2.)

Using an ESI source in both positive and negative ion monitoring mode, a single standard solution containing 11 pesticides (mass concentration: 0.2 mg/L) was continuously infused into the ion source at a flow rate of 7 μL/min. The abundance and stability of the parent ions, as well as the corresponding voltage value of the lens, were determined via full-scan mode. Then, a certain collision energy is applied to the determined parent ion to perform sub-ion fragment scanning. Two pairs of relatively high abundance and low interference fragment ions are selected for each compound as quantitative and qualitative ion pairs, respectively. The specific optimization parameters of the 11 pesticides are determined in Table 1, the TIC is shown in Figure 2, and the extracted ion chromatograms of each compound are shown in Figure 3.

### 2.2. Purification Optimization of Pre-Treatment

#### 2.2.1. Selection of Purification Agent Types

Seven purification agents commonly used in the QuEChERS method, namely GCB, Florisil, Alumina-N, MgSO_4_, PSA, C_18_, and MWNTs, were selected for experimental optimization to investigate the effects of different purification agents on the recovery rate of tested pesticides.

According to the results in Table 2, among the seven selected purification agents, the recovery rates of MgSO_4_, Florisil, and C_18_ all meet the requirements of GB/T 27404-2008 [15].

#### 2.2.2. Optimization of Purification Agents Ratio

Fruits and vegetables contain a high amount of water, vitamins, pigments, etc. Florisil is utilized to remove polar impurities and fatty acids, C_18_ is employed to adsorb fats, and MgSO_4_ serves to remove water. For further optimization, a combination of MgSO_4_ and Florisil was used to purify the extraction solution. We fixed the amount of MgSO_4_ at 25 mg per milliliter of extract, using 6 mL of extract solution as an example, and optimized the amount of Florisil. For this purpose, five different ratios were designed: (1) 30 mg Florisil + 150 mg MgSO_4_; (2) 90 mg Florisil + 150 mg MgSO_4_; (3) 150 mg Florisil + 150 mg MgSO_4_; (4) 210 mg Florisil + 150 mg MgSO_4_; (5) 270 mg Florisil + 150 mg MgSO_4_. Based on the results of pigment changes in the samples and the recovery rates of the compounds, after determining the optimal content of Florisil, we further optimized the ratio of MgSO_4_ by setting five different weight ratios: ① Florisil: MgSO_4_ = 1:0.5; ② Florisil: MgSO_4_ = 1:1; ③ Florisil: MgSO_4_ = 1:2; ④ Florisil: MgSO_4_ = 1:3; ⑤ Florisil: MgSO_4_ = 1:4. The relevant results can be found in Table 3.

Grains typically contain a certain quantity of starch, proteins, and lipids. Similar to the purification process applied to fruits and vegetables, in an effort to optimize the purification effect for grains, five different ratios of purification agents were designed and tested. Taking 6 mL of the grain extract solution as a representative example, we standardized the amount of MgSO_4_ at 25 mg per milliliter of extract. The five specific ratios of purification agents are as follows: (1) 80 mg Florisil + 150 mg MgSO_4_; (2) 150 mg Florisil + 150 mg MgSO_4_; (3) 280 mg Florisil + 150 mg MgSO_4_; (4) 400 mg Florisil + 150 mg MgSO_4_; (5) 450 mg Florisil + 150 mg MgSO_4_.The relevant results can be found in Table 4.

For oil crops, considering their high lipid content, the focus was mainly on comparing the purification effects of C_18_ and Florisil and exploring the impact of different sample weights. MgSO_4_ was used to remove water, and four different experimental conditions were set, with a fixed amount of MgSO_4_ at 150 mg for each group. The four conditions are as follows: ① 5 g of soybean sample + 150 mg MgSO_4_ + 150 mg C_18_; ② 2.0 g of soybean sample + 150 mg MgSO_4_ + 150 mg C_18_; ③ 5 g of soybean sample + 150 mg MgSO_4_ + 150 mg Florisil; ④ 2.0 g of soybean sample + 150 mg MgSO_4_ + 150 mg Florisil. The relevant results can be found in Table 4.

According to the results in Table 3, it can be clearly observed that when the amount of Florisil ranges from 5 to 25 mg per milliliter of the 6 mL purified solution (i.e., 30–150 mg Florisil for 6 mL of the solution), the recovery rate of each pesticide remains relatively stable. However, for pesticide T, the recovery rate decreases as the content of Florisil increases. When the Florisil content reaches a certain level, the recovery rates of cyhalopfop acid, pesticide Q, and pesticide H also show a downward trend. Regarding the influence of MgSO_4_ content, an increase in MgSO_4_ has little impact on the recovery rates of most pesticides. But for pesticide T, when the ratio of Florisil to MgSO_4_ exceeds 1:1, the recovery rate of T begins to decline. Therefore, the appropriate amount of Florisil is 5–25 mg per milliliter of the extraction solution. In this study, for a 6 mL extraction solution, purification was carried out by mixing 25 mg/mL of Florisil (150 mg in total) and 25 mg/mL of MgSO_4_ (150 mg in total).

According to Table 4, different ratios of purification agents have a relatively minor impact on the results of grains. To maintain the consistency of the method, we selected Scheme (2) as the ratio of purification agents for grains, that is, 25 mg of MgSO_4_ and 25 mg of Florisil are formulated per milliliter of the extraction solution. For oil crops, we chose Scheme (2) as the ratio of purification agents for oil crops. Specifically, for a 2 g sample weight, after extraction, 25 mg of MgSO_4_ and 25 mg of Florisil are formulated per milliliter of the extraction solution.

#### 2.2.3. Purification Optimization of Dark Vegetables

For dark vegetables, a small amount of GCB needs to be added to remove pigment interference. Using chives to do the experiment, an appropriate amount of GCB was selected to achieve the effect of color removal while ensuring that the recovery rate of each pesticide was within a good range. The design ratio of 6 mL extraction solution was as follows: ① 150 mg Florisil + 150 mg MgSO_4_ + 60 mg GCB; ② 150 mg Florisil + 150 mg MgSO_4_ + 90 mg GCB; ③ 150 mg Florisil + 150 mg MgSO_4_ + 120 mg GCB; ④ 150 mg Florisil + 150 mg MgSO_4_ + 150 mg GCB; ⑤ 150 mg Florisil + 150 mg MgSO_4_ + 180 mg GCB.

Experiments and measurements were conducted following the same pre-treatment procedures described in Section 3.4. The blank solution of the base standard was purified under the purification conditions of Scheme ①. The purification effects of different schemes were evaluated by analyzing their average recovery rates, and the results are presented in Table 5.

As shown in Table 5, with the increase in GCB dosage, the recovery rates of C, cyhalopfop acid, Q, H, and QPT all showed a downward trend. Among them, Q showed the most obvious downward trend. When 120 mg GCB was added to 6 mL of extraction solution, the recovery rate of Q was 82.1%. When the GCB dosage was increased to 180 mg, the pigments in the purified extraction solution were basically removed, But the recovery rate of Q was only 36.2%. It was recommended to use 150 mg Florisil + 150 mg MgSO_4_ + 120 mg GCB as the purification agent for chives extract, as it ensured good recovery rates for all 11 target substances and acceptable purification efficiency.

### 2.3. Detection and Quantification Limits of the Method

The blank value and the concentration of the spiked sample were quantitatively determined using the matrix standard curve. Subsequently, the weighted standard deviation (SD) was calculated according to Equation (1) below and then the LOD value was obtained by applying Equation (2) [16]. The LOD values for the five substrates of cabbage, pear, chives, wheat flour, and soybean oil are presented in Table 6, with R^2^ values above 0.993.

(1) $$SD=\sqrt{\frac{(m-1)SD_{A}^{2}+(n-1)SD_{B}^{2}}{m+n-2}}$$

LOD = 2 × t_0.05(f)_ × SD/S (2)

In Equation (1), “m” is the number of measurements for the blank sample, “n” is the number of repetitions for a certain added concentration, “SD_A_” is the standard deviation of the blank sample, and “SD_B_” is the standard deviation of a sample with added concentration. In Equation (2), t_0.05(f)_ can be obtained from the statistical table, and the sensitivity “S” can be estimated from the average recovery value and addition level. S = average sample concentration B/minimum addition concentration (the minimum addition concentration is generally the lowest point on the standard curve or the lowest concentration point that can peak).

In cabbage, pear chives, wheat flour, and soybean oil, the limit of quantification (LOQ) of the method is 0.005–0.01 mg/kg. The quantitative limit of this method can meet the detection requirements of the national standard limit, but for the setting of the quantitative limit, considering the addition of herbicides and metabolites, the influence of recovery rate, and the standard limit value, a too low quantitative limit was not set.

### 2.4. Accuracy and Precision of Methods

Concentrations of 0.01 mg/kg, 0.05 mg/kg, and 0.2 mg/kg were added to the blank cabbage samples. For the blank chive samples, concentrations of 0.01 mg/kg, 0.05 mg/kg, and 0.1 mg/kg were, respectively, added. Similarly, for the blank pear samples, concentrations of 0.01 mg/kg, 0.02 mg/kg, and 0.1 mg/kg were added. In the case of blank wheat flour samples and blank soybean oil samples, concentrations of 0.01 mg/kg, 0.05 mg/kg, and 0.2 mg/kg were added. The average recovery rate and RSD were quantitatively calculated using matrix-matched standards combined with an external standard method to evaluate the accuracy and precision of the method. The results are shown in Table 7. The results showed that in cabbage and chives, the recovery rates of 11 pesticides at three levels were 69.8~115%, and the RSD was 0.6~8.6%; the recovery rates of 11 pesticides in pear at three levels were 80.0~120%, and the RSD is 0.6~17%; the recovery rates of 11 pesticides in wheat flour at three levels were 73.6~116%, and the RSD was 1.4~19; the recovery rates of 11 pesticides in soybean oil at three levels were 83.5~117%, and the RSD was 1.8~10%, meeting the requirements of methodology (GB/T 27404-2008) [15].

### 2.5. Actual Sample Testing

**Figure 4:**
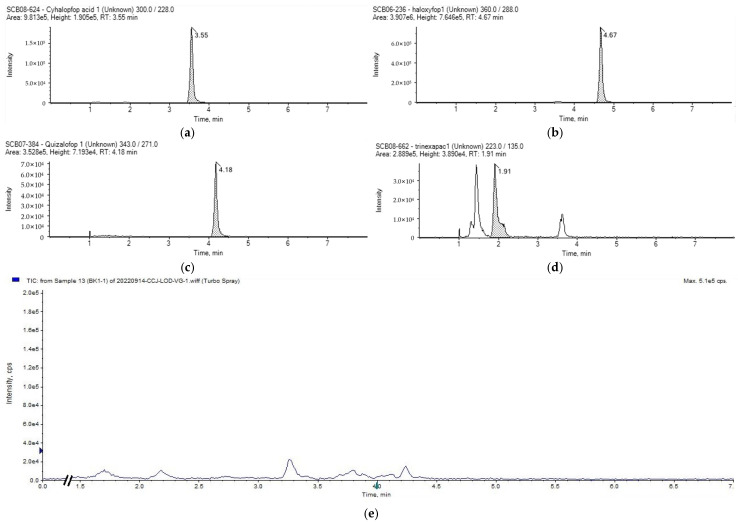
Quantitative extracted ion chromatograms of samples. (**a**) Cyhalofop acid in amaranth; (**b**) Haloxyfop in You Mai Cai; (**c**) Quizalofop in Hangzhou bok choy; (**d**) Trinexapac in Pak Choi seedlings; (**e**) blank cabbage matrix.

A total of 144 samples were collected from the Shanghai market, including 86 vegetables such as leafy vegetables, eggplants, brassicas, bulbs, rhizomes, and potatoes; 28 fruits such as nuts, drupes, and berries; 20 grains such as rice and wheat flours; and 10 edible oils such as peanut oils, soybean oils, and rapeseed oils. Use the established instrument and pre-treatment methods to detect actual samples, and use blank cabbage for addition and recovery as quality control during the process to ensure the accuracy and stability of the detection results. The mass spectrogram corresponding to some experimental results is presented in Figure 4. The target compounds were detected in 20 samples, with a total detection rate of 13.8%. As shown in Table 8, among the 20 detected samples, cyhalopfop acid, Q, T, and H were detected in Chinese little greens, Hangzhou cabbage, bok choy, amaranth, and peach; other herbicides and metabolites were not detected. According to the maximum residue limit requirements of the European Union for pesticides, the limit for CB in root vegetables is 0.02 mg/kg, and the limit for Q, T, and H in stem vegetables is 0.01 mg/kg [17]. The detection values of CB and CA are below the limit of 0.02 mg/kg, while the detection values of the other three herbicides and their residues are all above the limit of 0.01 mg/kg. However, the overall detected herbicide content is at a relatively low level, with only Q having a detection value exceeding 0.1 mg/kg, and the crops with detected herbicides are not registered. The actual sample testing results indicated that although herbicides were mainly applied to field crops, they may also be applied during the cultivation process of vegetables and fruits.

## 3. Materials and Methods

### 3.1. Instrumental Conditions

TQ5500 triple quadrupole mass spectrometer (AB Sciex, Framingham, MA, USA); Acquity (Waters, Milford, MA, USA) and Acquity UPLC BEH C18 column (2.1 × 100 mm, 1.7 μm, Waters, MA, USA); AL204-IC electronic analytical balance (METTLER TOLEDO, Zürich, Switzerland); Talboys digital display vortex oscillator (Anpu, Shanghai, China); high-speed desktop centrifuge (Sorvall ST 16R, Thermo Scientific, Waltham, MA, USA); ultra-pure water (18.2 MΩ·cm, Merck, Darmstadt, Germany).

### 3.2. Chemicals and Reagents

Acetonitrile (ACN, HPLC grade, Merck, Darmstadt, Germany); Formic acid (FA) and ammonium formate (HPLC grade, CNW, Düsseldorf, Germany); NaCl (analytical pure, Titan, Shanghai, China); Magnesium sulfate (MgSO_4_, analytical pure, CNW, Düsseldorf, Germany); Primary secondary amine (PSA, 40–63 μm. CNW, Düsseldorf, Germany); Graphitized carbon black (GCB, 37–119 μm. CNW, Düsseldorf, Germany); CNWBOND HC-C18 Bulk Sorbent (C_18_, 40–63 μm, CNW, Düsseldorf, Germany); Neutral alumina (Alumina-N, 46–149 μm. CNW, Düsseldorf, Germany); Multi-walled carbon nanotubes (MWNTs, 20–30 nm, Aladdin, Shanghai, China); and Florisil (149–209 μm, CNW, Düsseldorf, Germany).

Standard substances: Trinexapac-ethyl, Trinexapac, Haloxyfop-methyl (purity: 98.69%, 98.9%, 98.74%, Dr. Ehrenstorfer, Augsburg, Germany); Haloxyfop-P-methyl and Haloxyfop (purity: 96.7%; 98.2%, Anpu, Shanghai, China); Quizalofop-P-tefuryl, Quizalofop, and Clodinafop (purity: 96.6%, 98.0%, 99.0%, Alta, Tianjin, China); Clodinafop-propargyl (purity: 97%, Macklin, Shanghai, China); Cyhalofop-butyl and Cyhalopfop acid (purity: 98%, 96%, Yuanye, Shanghai, China).

### 3.3. Purification Agent Types Determination

A standard mixed solution of 11 pesticides with a mass concentration of 0.05 mg/L was prepared using an acetonitrile solution containing 0.1% formic acid by volume fraction. Separately, 6 mL of this standard mixed solution was taken and added to 150 mg of different purification agents, with each purification agent corresponding to a set of experiments. Each set of solutions was vortexed and mixed for 2 min and then centrifuged at 5000 r/min for 5 min. An amount of 2 mL of the supernatant from each set of solutions was aspirated using a disposable syringe and passed through a 0.22 μm organic filter membrane. The filtrates were measured according to the optimized conditions of the instrument. Three parallel tests were performed for each purification plan.

### 3.4. Ratio of Purification Agents

Cabbage was selected as the representative of vegetable matrices, pear was selected as the representative of fruit matrices, wheat flour as the representative of grain matrices, and soybean as the representative of oil–crop matrices. Using the blank solutions of these respective matrix representatives as solvents, a standard mixture of 11 pesticides with a specific concentration was prepared. The blank matrix solutions used for preparing the matrix standard curves of these three representative matrices were purified according to the conditions specified in Scheme (1). Subsequently, 6 mL of the prepared standard pesticide mixture was transferred into a centrifuge tube containing the aforementioned dispersive solid-phase extraction (dSPE) purification agent. The mixture was allowed to stand for 2 h, then vortex-mixed at a speed of 2500 r/min for 2 min, followed by centrifugation at 5000 r/min for 5 min. After that, the supernatant was passed through a 0.22 μm organic filter membrane, and the resulting filtrate was measured according to the optimized working conditions of the instrument.

### 3.5. Preparation of Standard Solutions

Weigh 10 mg (±0.1 mg) of each standard substance separately, which was diluted to 10 mL with acetonitrile to prepare a standard stock solution with a concentration of about 1000 mg/L. The standard stock solution was stored at −20 °C. Take an appropriate amount of single standard storage solution, prepare a mixed standard solution containing 11 pesticides with a concentration of 10 mg/L, and store at −20 °C in the dark. Among them, the instrument response values of the two pesticides, trinexapac and cyhalofop-butyl, were relatively low, so their concentrations in the mixed standard were increased to 50 mg/L and 100 mg/L, respectively. Take an appropriate amount of 11 mixed standard solutions of pesticides and prepare series blank matrix mixed standard solution of cabbage, pear, chives, wheat flour, and soybean oil with concentrations of 0.0005 mg/L, 0.001 mg/L, 0.002. mg/L, 0.005 mg/L, 0.010 mg/L, 0.020 mg/L, and 0.050 mg/L, used for detection and analysis [18,19].

### 3.6. Sample Preparation and QuEChERS Procedure

Take 600–800 g cabbage, pear, chives, wheat flour, or soybean oil samples using the quartering method and prepare them into a homogenate or powder (powder could be able to pass through 425 μm standard mesh sieve), place in a clean container, and store in a −18 °C refrigerator for later use.

QuEChERS procedure of fruits and vegetables: Weigh 10 g (±0.1 g) of the sample into a 50 mL centrifuge tube, add 10 mL 0.1% formic acid acetonitrile, vortex for 10 min, add 4 g NaCl, and centrifuge at 5000 r/min for 5 min. Quantitatively transfer the supernatant into a plastic centrifuge tube pre-filled with water-removing agent and purification materials (specifically, 25 mg/L of MgSO_4_ and 25 mg/L of Florisil of the extraction solution). If the sample is dark vegetables, add 120 mg GCB for color removal. Vortex at 2500 r/min for 2 min, centrifuge at 5000 r/min for 5 min, and pass through 0.22 μm filter membrane.

QuEChERS procedure of grains: Weigh 5 g (±0.1 g) of the sample into a 50 mL centrifuge tube, add 10 mL 0.1% formic acid water by volume fraction, and vortex and mix for 10 min. Add 15 mL 0.1% formic acid and acetonitrile solution, vortex for 10 min, add 4 g NaCl, and centrifuge at 5000 r/min for 5 min. Quantitatively transfer the supernatant into a plastic centrifuge tube pre-filled with water-removing agent and purification materials (specifically, 25 mg/L of MgSO_4_ and 25 mg/L of Florisil of the extraction solution). Vortex for 2 min. Centrifuge at 5000 r/min for 5 min and pass through 0.22 μm filter membrane.

QuEChERS procedure of oil crops: Weigh 2 g (±0.1 g) of the sample into a 50 mL centrifuge tube. Then, add 5 mL of 0.1% formic acid water by volume fraction vortex and mix it for 1 min to ensure thorough dispersion. Then, introduce 10 mL of a 0.1% formic acid acetonitrile solution by volume fraction and vortex-mix for 10 min to facilitate full-scale extraction. Next, add 4 g of MgSO_4_ to the mixture and subject it to centrifugation at 5000 r/min for 5 min to separate the phases. Quantitatively transfer the supernatant into a plastic centrifuge tube pre-filled with water-removing agent and purification materials (specifically, 25 mg of MgSO_4_ and 25 mg of C_18_ are utilized per milliliter of the extraction solution). Vortex-mix the new mixture for 2 min to achieve uniform distribution. Subsequently, centrifuge this mixture at 5000 r/min for 5 min to further separate the components. Finally, aspirate the supernatant and pass it through a microporous filter membrane, making it ready for subsequent determination.

### 3.7. UPLC-MS/MS Conditions

Chromatographic column was Waters Acquity UPLC BEH C18, mobile phase A was acetonitrile, mobile phase B was 2 mmol/L ammonium formate solution with a volume fraction of 0.1% formic acid. Chromatographic column temperature was at 35 °C; injection volume was 2 μL; flow rate was 0.3 mL/min; and gradient elution was as follows: from 0 to 1 min, the mobile phase A remained unchanged at 40%; from 1 to 6 min, A gradually transformed into 90%; from 6 to 7 min, A gradually transformed into 40%; from 7 to 8 min, A remained unchanged at 40%.

We referred to the relevant mass spectrometry conditions in references [20,21,22,23], and made slight improvements according to the actual situation of the instrument, using ESI ion source, positive and negative ion scanning, and multi-reaction monitoring mode (MRM). Ion Source Gas1 (GS1) was 50 psi, ion Source Gas2 (GS2) was 40 psi, the curtain gas was 35 psi, and the collision gas was 8 psi. The ion spray voltage was 5500 V/−5500 V, the ion source temperature was 250 °C, and the mass spectrum parameters of pesticides are shown in Table 1.

## 4. Conclusions

In this study, a method for the simultaneous determination of six herbicides and their metabolites by using the QuEChERS procedure combined with high-performance liquid chromatography–tandem mass spectrometry was established. Acetonitrile with a volume fraction of 0.1% formic acid was used as the extraction solution. An amount of 6 mL extraction solution was purified with 150 mg Florisil and 150 mg MgSO_4_. For darker colored matrices, an additional 120 mg GCB was added for color removal, which was the extraction and purification step of this method. After multiple experimental verifications, this method had high sensitivity, good precision, and fast separation speed, which could meet the requirements for residual detection of six herbicides and their metabolites in fruits, vegetables, grains, and edible oils; this method also had good application prospects.

## Figures and Tables

**Table 1:** MS detection parameters of the 11 pesticides.

Compound Name	Parent Ion (*m*/*z*)	Product Ion (*m*/*z*)	Collision Energy (eV)	Declustering Potential (V)	CAS
C	310	238 */218	−19/−29	−80	114420-56-3
CA	300	228 */208	−21/−28	−82	122008-78-0
Q	343	271 */243	−19/−35	−70	76578-12-6
T	223	135 */179	−21/−20	−80	104273-73-6
H	360	288 */252	−17/−34	−97	69806-34-4
CB	375	256 */120	22/44	85	122008-85-9
HPM	376	288 */91	35/36	133	72619-32-0
TE	253	69 */207	30/17	97	95266-40-3
QPT	429.2	299.3 */270.9	31/38	112	200509-41-7
HM	376	316.1 */288.1	22/35	162	69806-40-2
CP	350	266.2 */91	19/36	166	105512-06-9

* is a qualitative ion.

**Table 2:** Effect of different purifiers on the recovery of pesticides (%).

Compound Name	GCB	Florisil	Alumina-N	MgSO_4_	PSA	C_18_	MWNTs
C	93.3	96.1	89.3	103	41.8	101	40.4
CA	93.9	101	85.4	98.2	39.7	104	66.8
Q	6.17	95.3	75.3	97.2	39.2	98.6	0.43
T	95.9	101	21.3	100	54.5	102	91.2
H	92.8	99.6	87.1	99.1	43.5	101	94.6
CB	90.5	93.2	96.3	89.5	98.8	85.9	96.3
HPM	96.6	97.9	106	101	93.1	91.0	81.5
TE	95.8	96.8	65.4	103	94.4	87.6	105
QPT	3.80	101	84.5	109	93.6	95.4	0.99
HM	101	99.0	113	102	101	98.9	79.4
CP	91.0	98.8	107	105	101	99.4	78.3

**Table 3:** Results in fruits and vegetables.

Compound Name	Optimization of Florisil					Optimization of MgSO_4_				
	(1)	(2)	(3)	(4)	(5)	①	②	③	④	⑤
C	93.9	95.2	95.6	106	88.5	109	114	111	113	115
CA	99.5	98.5	99.0	94.9	95.5	105	103	107	103	105
Q	96.9	91.9	93.7	93.0	87.6	100	104	98.7	103	103
T	97.3	84.6	66.7	49.3	30.4	62.6	69.0	70.3	60.4	56.9
H	97.7	88.3	93.0	88.9	86.0	104	106	104	106	109
CB	108	108	136	118	114	108	105	110	111	103
HPM	99.8	99.0	95.1	94.4	95.0	102	101	106	107	111
TE	98.2	99.5	96.9	97.6	94.7	95.2	95.6	93.6	97.8	93.4
QPT	100	95.7	91.7	94.4	95.1	107	113	106	109	114
HM	100	99.2	99.0	97.7	97.3	102	103	104	109	108
CP	98.7	94.6	96.9	96.9	93.7	104	106	97.5	107	108

**Table 4:** Results in grains and oil crops.

Compound Name	Grains					Oil Crops			
	(1)	(2)	(3)	(4)	(5)	(1)	(2)	(3)	(4)
C	101	99	101	97	101	77.8	89.0	91.3	73.0
CA	102	100	102	96	100	94.7	98.8	73.7	92.9
Q	100	99	101	100	100	70.2	82.9	70.0	84.7
T	111	113	102	75	99	66.5	86.6	19.2	67.8
H	102	102	99	98	99	79.1	87.9	75.7	87.1
CB	110	90	94	102	105	68.5	78.3	73.1	74.8
HPM	104	94	96	104	103	76.2	88.8	79.4	77.5
TE	98	101	104	96	101	92.9	96.0	74.0	100.7
QPT	91	94	101	106	108	77.8	89.1	76.6	84.8
HM	100	93	97	108	103	80.6	91.2	78.2	87.8
CP	91	95	98	106	110	73.0	91.9	77.8	91.5

**Table 5:** The effect of different content of GCB on the recovery rate of pesticides (%).

Compound Name	① 60 mg	② 90 mg	③ 120 mg	④ 150 mg	⑤ 180 mg
C	100	107	98.1	96.2	96.1
CA	101	93.1	96.2	93.8	88.6
Q	113	95.5	82.1	63.5	36.2
T	104	112	112	104	100
H	89.9	91.7	90.1	90.2	83.7
CB	114	109	114	111	116
HPM	99.2	97.8	99.6	105	110
TE	97.5	95.6	99.7	98.5	100
QPT	97.4	95.8	92.0	83.2	67.5
HM	101	105	102	112	114
CP	97.9	97.9	102	101	110

**Table 6:** Linear range and LOD of standard curve of cabbage, pear, chives, wheat flour, and soybean oil.

Compound Name	Linear Range (mg/L)	Cabbage LOD (mg/kg)	Pear LOD (mg/kg)	Chives LOD (mg/kg)	Wheat Flour LOD (mg/kg)	Soybean Oil LOD (mg/kg)
C	0.0005~0.05	0.0003	0.0002	0.001	0.001	0.0003
CA	0.0005~0.05	0.0001	0.0004	0.0001	0.001	0.0005
Q	0.0005~0.05	0.0004	0.0001	0.0005	0.0007	0.0005
T	0.0025~0.25	0.001	0.0006	0.003	0.008	0.003
H	0.0005~0.05	0.0002	0.0008	0.0001	0.001	0.0004
CB	0.005~0.5	0.008	0.004	0.005	0.01	0.005
HPM	0.0005~0.05	0.0001	0.0001	0.0005	0.0008	0.0002
TE	0.0005~0.05	0.0001	0.0002	0.0006	0.01	0.0006
QPT	0.0005~0.05	0.0001	0.0002	0.0004	0.001	0.0005
HM	0.0005~0.05	0.0001	0.0001	0.0003	0.0005	0.0004
CP	0.0005~0.05	0.0001	0.0001	0.0001	0.0005	0.0003

**Table 7:** Average recoveries and relative standard deviations (RSD) of 11 pesticides in cabbage, pear, chives, wheat flour, and soybean oil (n = 6).

Compound Name		Cabbage			Pear			Chives			Wheat Flour			Soybean Oil		
		0.01 mg/kg	0.05 mg/kg	0.2 mg/kg	0.01 mg/kg	0.02 mg/kg	0.1 mg/kg	0.01 mg/kg	0.05 mg/kg	0.1 mg/kg	0.01 mg/kg	0.05 mg/kg	0.2 mg/kg	0.01 mg/kg	0.05 mg/kg	0.2 mg/kg
C	Recovery/%	105	108	98.4	110	112	113	97.3	92.3	94.9	86.6	104	102	105	101	98.6
	RSD/%	1.8	1.9	3.6	7.0	7.8	1.5	3.8	4.0	3.5	2.7	2.5	2.3	5.7	3.0	2.2
CA	Recovery/%	110	113	105	107	107	120	99.3	104	106	105	103	98.9	105	103	101
	RSD/%	3.1	1.5	3.4	9.4	3.3	0.8	6.8	5.0	4.3	6.1	2.5	3.5	3.4	4.8	2.2
Q	Recovery/%	106	111	105	107	115	118	69.8	70.3	75.6	106	103	99.0	105	98.5	98.0
	RSD/%	4.6	1.7	4.4	6.8	5.0	1.2	5.5	7.5	5.9	8.1	2.2	2.0	4.6	3.8	2.8
T	Recovery/%	103	105	105	84.3	97.1	100	77.4	76.4	71.2	93.1	93.2	90.3	110	101	98.2
	RSD/%	3.8	2.6	2.3	7.8	4.8	3.2	7.0	6.8	8.1	6.2	3.0	4.3	3.4	3.8	2.6
H	Recovery/%	110	111	110	109	118	119	98.9	99.6	102	116	110	107	107	101	100
	RSD/%	4.1	2.9	2.4	7.5	5.2	1.8	6.2	5.0	3.6	5.4	1.6	3.0	3.6	2.5	3.3
CB	Recovery/%	99.9	97.9	101	89.2	103	80.0	108	109	114	73.6	112	99.2	107	117	103
	RSD/%	2.6	3.7	3.1	6.3	9.2	3.9	8.6	8.5	4.2	18	3.7	8.3	10	1.8	7.0
HPM	Recovery/%	105	103	89.4	107	117	119	111	114	114	105	108	98.2	102	100	93.6
	RSD/%	5.9	1.9	2.8	6.7	9.5	3.9	2.8	3.5	3.0	9.3	1.8	5.6	6.0	2.0	2.4
TE	Recovery/%	110	112	102	109	114	120	98.4	103	99.7	104	105	106	83.5	85.6	85.0
	RSD/%	3.8	1.8	1.7	6.1	6.7	0.6	6.9	3.6	3.2	4.0	2.3	1.5	9.6	3.1	2.5
QPT	Recovery/%	112	108	98.4	105	113	112	103	106	106	113	111	106	97.0	91.6	90.0
	RSD/%	4.7	2.4	2.2	5.1	11	13	4.7	4.1	4.6	5.2	1.4	4.7	3.0	2.3	2.9
HM	Recovery/%	107	97.0	90.3	108	115	116	109	115	115	106	104	93.4	103	97.9	93.1
	RSD/%	5.3	3.2	4.9	5.9	11	8.0	4.1	2.5	2.5	6.7	6.7	5.5	3.1	2.7	2.0
CP	Recovery/%	109	110	109	106	115	109	106	108	115	116	108	99.3	104	96.7	95.9
	RSD/%	5.3	2.0	2.0	8.7	17	15	6.0	3.8	1.8	19	9.6	9.9	3.0	2.7	2.9

**Table 8:** Analysis of herbicide detection results in detected samples (mg/kg).

Compound Name	Detected Quantity	Average Value	Maximum
CA	1	0.049	0.049
Q	2	0.096	0.172
T	17	0.021	0.048
H	3	0.027	0.055

## Data Availability

The original contributions presented in this study are included in the article material. Further inquiries can be directed to the corresponding author(s).

## Abbreviations

CP, Clodinafop-propargyl; C, Clodinafop; QPT, Quizalofop-P-tefuryl; Q, Quizalofop; H, Haloxyfop; HM, Haloxyfop-methyl; HPM, Haloxyfop-p-methyl; CB, Cyhalofop-butyl; CA, Cyhalofop acid; TE, Trinexapac-ethyl; T, Trinexapac; GCB, Graphitized carbon black; PSA, Ethylenediamine-N-propylsilane; MWNTs, Multi-walled carbon nanotubes; C_18_, CNWBOND HC-C18 Bulk Sorbent; QuEChERs, quick, easy, cheap, effective, rugged, and safe; dSPE, dispersive solid-phase extraction cleanup; TIC, Total Ion Chromatogram; MRLs, Maximum Residue Limits; RSD, relative standard deviation; LOD, limit of detection; ESI, electrospray ionization.
